# ICTV Virus Taxonomy Profile: Pleolipoviridae 2022

**DOI:** 10.1099/jgv.0.001793

**Published:** 2022-11-14

**Authors:** Ying Liu, Mike Dyall-Smith, Hanna M. Oksanen

**Affiliations:** 1 Archaeal Virology Unit, Institut Pasteur, Université Paris Cité, CNRS UMR6047, Paris, France; 2 Computational Biology Group, Max Planck Institute of Biochemistry, Martinsried, Germany; 3 Veterinary Biosciences, Faculty of Veterinary and Agricultural Sciences, University of Melbourne, Parkville, Australia; 4 Molecular and Integrative Biosciences Research Programme, Faculty of Biological and Environmental Sciences, University of Helsinki, Helsinki, Finland

**Keywords:** ICTV Report, *Pleolipoviridae *, taxonomy, archaeal virus

## Abstract

Members of the family *Pleolipoviridae* are pseudo-spherical and pleomorphic archaeal viruses composed of a membrane vesicle, which encloses a DNA genome. The genome is either circular ssDNA or dsDNA, or linear dsDNA molecules of approximately 7 to 17 kilonucleotides or kbp. Typically, virions contain a single type of transmembrane spike protein at the envelope and a single type of membrane protein, which is embedded in the envelope and located in the internal side of the membrane. All viruses infect extremely halophilic archaea in the class Halobacteria (phylum Euryarchaeota). Pleolipoviruses have a narrow host range and a persistent, non-lytic life cycle. Some viruses are temperate and can integrate into the host chromosome. This is a summary of the International Committee on Taxonomy of Viruses (ICTV) Report on the family *Pleolipoviridae*, which is available at ictv.global/report/pleolipoviridae.

## Virion

**Fig. 1.**
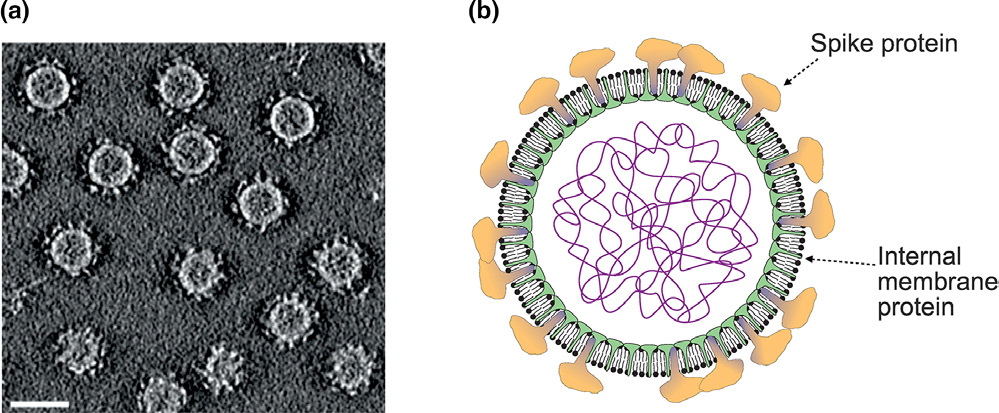
Morphology of pleolipovirus virions. (**a**) A slice through a three-dimensional cryo-electron microscopy tomogram of Halorubrum pleomorphic virus 6 reconstructed from tilt series data. Reproduced with permission from [3] under Creative commons license 4.0. Bar, 40 nm. (**b**) Schematic of the pleolipovirus virion.

Virions are enveloped pleomorphic membrane vesicles of 40–70 nm diameter (Table 1, Fig. 1a) with one or two types of major proteins forming spikes and one or two as internal membrane proteins (Fig. 1b) [1,2]. The fusion-inducing spike protein has a unique V-shaped fold and belongs to a new structural class of fusion proteins [3]. Virions lack a capsid or nucleocapsid.

**Table 1.** Characteristics of members of the family *Pleolipoviridae*

Example	Halorubrum pleomorphic virus 1 (HRPV-1; FJ685651), species *Alphapleolipovirus HRPV1*, genus *Alphapleolipovirus*
Virion	Enveloped, pseudo-spherical and pleomorphic virions (diameter 40–70 nm), typically with a single type of spike protein at the envelope and a single type of internal membrane protein embedded in the envelope
Genome	Circular ssDNA, circular dsDNA or linear dsDNA, 7–17 kilonucleotides or kbp
Replication	Possibly rolling-circle replication for circular molecules; protein-primed replication for linear molecules
Translation	Prokaryotic translation using viral mRNA and host ribosomes
Host range	Archaea; euryarchaeal *Halorubrum*, *Haloarcula*, *Halogeometricum* or *Natrinema* strains
Taxonomy	Realm *Monodnaviria*, kingdom *Trapavirae*, phylum *Saleviricota*, class *Huolimaviricetes*, order *Haloruvirales*: three genera *Alphapleolipovirus*, *Betapleolipovirus* and *Gammapleolipovirus*

## Genome

**Fig. 2.**
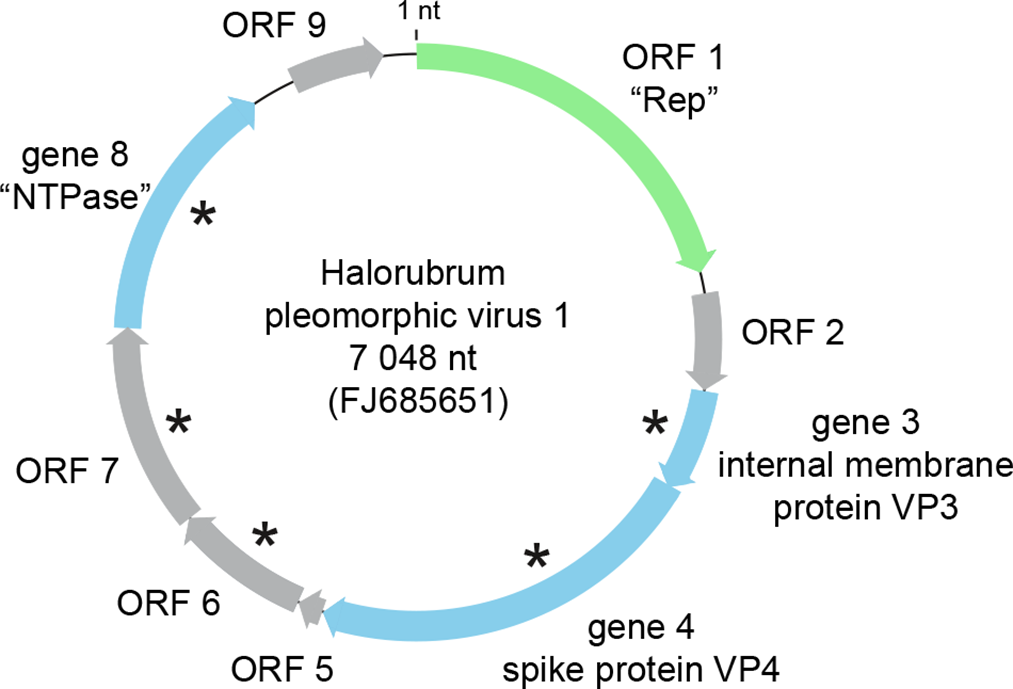
Genome organization of Halorubrum pleomorphic virus 1. Blue - structural proteins (NTPase - nucleoside triphosphate hydrolase); green - ORF1 encoding a putative replication initiation protein (Rep); Grey - other ORFs; asterisk - core gene.

Virus genomes are circular ssDNA of 7–11 kilonucleotides (Fig. 2), circular dsDNA of 8–17 kbp or linear dsDNA of 16 kbp. Members of the genus *Alphapleolipovirus* have circular ssDNA or dsDNA genomes, members of the genus *Betapleolipovirus* have circular dsDNA genomes with single-stranded discontinuities, and members of the genus *Gammapleolipovirus* have linear dsDNA genomes [4,7]. A cluster of five genes/ORFs is conserved among members of the family (Halorubrum pleomorphic virus 1 genes *3*, *4* and *8*, ORFs 6 and 7). The cluster includes genes encoding a spike and an internal membrane protein, as well as an ORF encoding a putative NTPase (Fig. 2). Members of the genus *Gammapleolipovirus* are predicted to encode a putative type B DNA-dependent DNA polymerase [6]. The genome ends bear terminal proteins.

## Replication

Pleolipoviruses are non-lytic and most likely enter cells by membrane fusion [3]. Either rolling-circle or protein-primed genome replication may be used. The transcription of His2 virus has been reported using a microarray approach [8]. Progeny virions exit host cells continuously, retarding host growth with concurrent unselective lipid acquisition, indicating that virions bud through the cell membrane [2]. Some viruses are temperate and can integrate in the host chromosome [7].

## Taxonomy

Current taxonomy: ictv.global/taxonomy. The family *Pleolipoviridae* includes three genera: *Alphapleolipovirus*, *Betapleolipovirus* and *Gammapleolipovirus* [1]. The genera are identified by gene content and well-supported monophyletic groups based on phylogenomic analysis of whole-genome sequences. Members of the genus *Alphapleolipovirus* share an ORF encoding a rolling circle replication initiation protein (RCR Rep). Betapleolipoviruses share two ORFs encoding proteins of unknown function (e.g. Halorubrum pleomorphic virus 3 ORFs 6 and 9). Gammapleolipovirus genomes have a gene encoding a putative type B DNA polymerase [7]. Viruses with genomes that differ by more than 5 % in nucleotide sequence are assigned to different species.

## Resources

Full ICTV Report on the family *Pleolipoviridae*: www.ictv.global/report/pleolipoviridae.
